# Effect of MBE growth conditions on GaAsBi photoluminescence lineshape and localised state filling

**DOI:** 10.1038/s41598-021-04477-0

**Published:** 2022-01-17

**Authors:** N. J. Bailey, T. B. O. Rockett, S. Flores, D. F. Reyes, J. P. R. David, R. D. Richards

**Affiliations:** 1 Department of Electronic and Electrical Engineering, University of Sheffield, Sheffield, UK; 2 University Research Institute on Electron Microscopy & Materials, (IMEYMAT), Universidad de Cádiz, Puerto Real, 11510 Cádiz, Spain

**Keywords:** Materials for devices, Materials for optics

## Abstract

A series of gallium arsenide bismide device layers covering a range of growth conditions are thoroughly probed by low-temperature, power-dependent photoluminescence measurements. The photoluminescence data is modelled using a localised state profile consisting of two Gaussians. Good agreement with the raw data is achieved for all layers whilst fixing the standard deviation values of the two Gaussians and constraining the band gap using X-ray diffraction data. The effects of growth temperature and bismuth beam equivalent pressure on the localised state distributions, and other model variables, are both shown to be linked to emission linewidth and device properties. It is concluded that bismuth rich surface conditions are preferable during growth in order to produce the narrowest emission linewidths with this material. These results also show how the growth mode of a gallium arsenide bismide layer can be inferred ex-situ from low-temperature photoluminescence measurements.

## Introduction

Gallium arsenide bismide (GaAsBi) is a material which has been of interest for optoelectronic devices in both telecommunication and solar photovoltaic applications for over a decade^1–4^. It has been shown that the incorporation of bismuth (Bi) into gallium arsenide (GaAs) causes a significant reduction of the band gap, starting at ~ 80 meV/% Bi^5^, which is a greater per-unit reduction than produced by incorporating indium or antimony. Also, due to the large spin orbit splitting enhancement caused by Bi; it may be possible to suppress CSHS Auger recombination at Bi contents ≥ 10%^6^, which is of interest for producing more efficient telecoms laser devices^2^. It has recently been shown that incorporation of Bi also leads to a decrease in the hole ionisation coefficient in avalanche photodiodes^7^. This indicates it is a promising material for novel, low noise infra-red detectors, providing the saturation currents can be minimised.

One complication of growing GaAsBi is that low growth temperatures, below ~ 400 °C, are required to facilitate Bi incorporation^8,9^ and these low growth temperatures can affect the material quality due to the formation of growth defects such as arsenic (As) antisites^10,11^. The incorporation of Bi also leads to the formation of a localised density of states (LDOS) above the valence band^12,13^. These states can be indirectly observed through an “s” shaped deviation from the standard Varshni behaviour of temperature dependent photoluminescence (PL) measurements^13,14^. Suppression of this effect through p-doping has been displayed and was used to estimate that the proportion of incorporated Bi atoms which contribute to these localised states is approximately 0.2%^15^ and temperature dependent luminescence studies have indicated that these states exist up to around 90 meV above the valence band^15,16^.

There have been multiple accounts in the literature with varying agreement on how the localised states are distributed in GaAsBi. Imhof et al*.*^17^ applied a kinetic Monte Carlo model to the PL intensity, full-width-half-maximum (FWHM) and Stokes shift of a bulk GaAsBi structure. In this model a Gaussian and an exponential distribution were used to represent alloy fluctuation and bismuth clustering respectively. Valkovskii et al*.*^18^ also used a two-scale approach in a carrier hopping model to fit the Stokes shift and FWHM of GaAsBi. Gogineni et al*.*^19^ observed two exponential distributions through Urbach fitting of power dependent and temperature dependent PL measurements on a GaAsBi quantum well layer. Shakfa et al*.*^20^ tested four combinations of exponential and Gaussian distributions in a two-scale fitting of thermal quenching of PL for several GaAsBi layers and found all to be mathematically viable although a Gaussian distribution was noted to be more physically realistic for deeper states. In 2018, Wilson et al*.*^21^ produced a state filling model to fit the low-temperature power dependant PL of a GaAsBi layer, which convolves a profile for the LDOS below the bandgap with a Gaussian distribution representing exciton emission broadening due to material defects. Here it was found that a Gaussian distribution better represented the emission from the GaAsBi layer than an exponential distribution. More recently, Yan et al*.*^22^ extracted three energy features using second-order derivative analysis on low temperature PL from two GaAsBi layers. These features were attributed to band-tail states rather than band-band transitions, which has also been observed elsewhere^23^.

Whilst the layers which have been studied in the literature were produced using various growth conditions, few reports have studied a coordinated series of layers to see how the growth conditions affect the formation of these states. The benefit of such a contained study is that it removes discrepancy of temperature and flux calibrations between different growth laboratories, which are prohibitively difficult to account for reliably and have a significant impact on material properties in the dilute bismide material system^24,25^. A recent study on localised state formation which covers a comprehensive series of layers is from Kakuyama et al*.*^26^, who studied six layers from a series with different growth temperatures and Bi fluxes. These layers were grown as p-i-n devices on both n-type and p-type substrates to facilitate both electrical and optical characterisation. Using current–voltage, photocurrent and PL measurements they found that higher growth temperatures suppressed localised state formation which was evidenced by a reduction in the Urbach energy, PL FWHM and the deviation from the predicted Varshni curve.

In this work we adapt the model presented by Wilson et al*.*^21^ and apply it to describe the low temperature PL of a large series of GaAsBi devices which cover a range of growth temperatures and bismuth beam equivalent pressures (BEPs).

## Experimental setup

The devices studied in this paper are described in Table 1. Bi contents of the layers are calculated from X-ray diffraction (XRD) simulations using a gallium bismide lattice parameter of 6.28 Å^27^ and from room temperature PL measurements. This series contains two subseries, one varying growth temperature (indicated by increasing ‘G’ value) and one varying Bi BEP (indicated by increasing ‘B’ value) with one of devices (G2B3) belonging to both subseries. The growth of these devices has already been discussed elsewhere^25^ so will only be briefly reviewed here.

**Table 1:** Growth details for the GaAsBi devices^25^.

Layer name	GaAsBi growth temperature (°C)	Bi BEP (× 10^–7^ mbar)	Bi content from PL/XRD (% Bi)
G1B3	355	1.06	3.6/3.51
G2B3	375	1.06	3.2/3.25
G3B3	385	1.06	2.7/2.82
G4B3	395	1.06	2.2/2.19
G5B3	405	1.06	1.3/1.37
G2B1	375	0.5	1.2/1.31
G2B2	375	0.76	2.2/2.25
G2B4	375	1.5	4.0/4.12
G2B5	375	2.12	5.3/5.37

The devices were grown on n-doped GaAs (001) substrates in an Omicron MBE-STM system. The growth rate was calibrated at the end of the buffer growth using reflected high energy electron diffraction (RHEED) to give 0.6 ± 0.01 ML/s. The devices consisted of a 300 nm n-type GaAs buffer grown at 577 °C followed by 100 nm of nominally undoped GaAsBi and 10 nm of undoped GaAs both grown at a reduced temperature. As_4_ was used for the growth of the i-region material which required a 20 min growth pause before and after the i-region growth to facilitate the arsenic cracker temperature change. Finally, a 300 nm p-type GaAs cap and < 10 nm p + GaAs contact layer were deposited at 577 °C. The temperatures quoted for growth were calibrated using RHEED observations of reconstruction transitions at several different temperatures. Further detail on this procedure can be found elsewhere^27^. For PL characterisation, the devices were placed in a closed-loop He Cryostat and cooled to 30 K. A chopped 532 nm diode pumped solid state laser was used for optical pumping and a lock-in amplifier connected to a liquid nitrogen cooled Germanium photodetector and Horiba monochromator were used for measuring the output PL spectra. An excitation power range of 30–900 mW was used with a 33% duty cycle and 3 ms period to reduce sample heating. 30 K was selected for the low-temperature measurements in order to maintain continuity with the previous work using this model^21^ and to ensure excited carriers dropped to the deepest localised states^28^. Some luminescence from the doped substrate was visible in the raw PL data for layers G1B3, G2B5, G2B3 and G2B4 which were grown with the lowest temperatures and highest Bi fluxes. This was removed by subtracting a scaled PL spectrum from an epi-ready doped substrate. Fittings to the corrected data for these layers will be presented with uncorrected fittings available in the supplementary material. Energy dispersive X-ray spectroscopy (EDX) measurements performed in scanning transmission electron microscope mode (STEM) were also taken on a set of GaAsBi test structures (detailed in the supplementary material) for comparison of observed Bi fluctuations. STEM was performed using a double aberration corrected FEI Titan3 Cubed Themis operated at 200 kV. EDX mapping was accomplished with four embedded Bruker bd-4 sx detectors using ChemiSTEM technology. Cross sectional TEM samples were prepared at [110] pole axis by conventional techniques.

Preliminary results for this work were obtained using a direct reproduction of the Wilson et al*.* model. This was unable to fit the majority of layers, particularly in the low energy range of the spectra which displayed significant shoulder peaks in several layers—something not seen in the layer studied by Wilson et al.^21^. The model was therefore adjusted to take account of a non-monotonous density of states.

**Figure 1:**
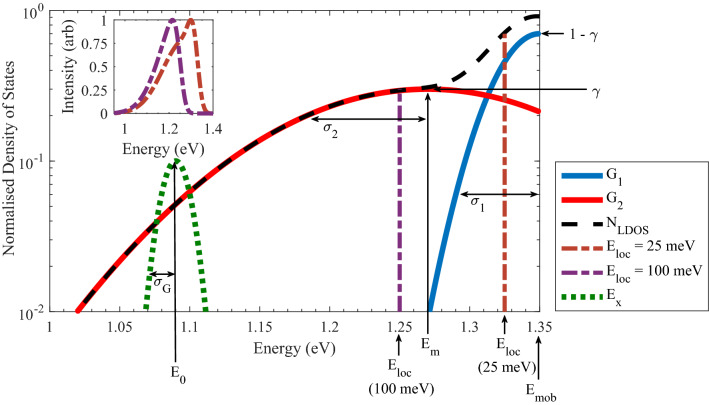
Example LDOS profile produced from Eq. (1). The overall profile (dashed line) is a summation of G1 and G2 (solid lines) which are produced from Eq. (2). Arrows have been added to indicate the meaning of each of the parameters in Eq. (1). Example localisation energies (dot-dashed lines) have been added to indicate the effect of N_LDOS_ on the model output PL (inset) after being convolved with E_x_ (dotted green line).

The model describes the PL lineshape as a convolution of a density of localised states with respect to energy (N_LDOS_)—as expressed in Eq. (1)—with an exciton emission profile (E_x_). N_LDOS_ is assumed to be partially filled, with all states up to a given energy occupied, and all states above empty. The energy up to which the states are filled is denoted as E_max_ = E_mob_ − E_loc_. Theoretically the model is evaluated from 0 eV to E_max_ but for practical reasons the minimum energy for which the model was evaluated, E_min_, was taken as the lowest energy measured from all the PL spectra for a given device where the model becomes statistically significant. A graphical illustration of the model is displayed in Fig. 1 using E_mob_ = 1.35 eV, E_m_ = 1.27 eV, γ = 0.3, σ_1_ = 0.027 eV and σ_2_ = 0.096 eV.

1 $${N}_{LDOS}(E)=\left[{\left(1-\gamma \right)\times G}_{1}\left(E,{E}_{mob},{\sigma }_{1}\right)\right]+\left[\gamma \times {G}_{2}(E,{E}_{m},{\sigma }_{2})\right]$$

Previously, Gaussian and exponential functions have been used to model localised state distributions. Here we have selected two Gaussian distributions scaled by a mixing parameter, γ to represent N_LDOS_. Gaussian distributions were selected as they have been found to produce accurate fits in previous studies and were deemed to be the most physically realistic^17,20,21^. Each distribution was defined by a centre energy (E_mob/m_) and standard deviation (σ_1/2_). Prior to summation via γ they were each scaled to a unity maximum, this was done to ensure the impact of γ was consistent regardless of intensity scaling by the standard deviation values. The G_1_ distribution was centred at the band edge estimated from XRD measurements, E_mob_, and accounts for band edge perturbations caused by alloy disorder^17^. G_2_ represents deeper clustering effects, and peaks at energy E_m_, which was allowed to take any value. G_1_ and G_2_ are expressed in generalised terms as:

2 $${G}_{1/2}(E,{E}_{mob/m},{\sigma }_{1/2})=\frac{1}{\sqrt{2\pi }\sigma }\times {e}^{-\frac{{\left(E-{E}_{mob/m}\right)}^{2}}{2{\sigma }_{1/2 }^{2}}}$$

The final piece of the model is the exciton profile, E_x_, which was assumed to also take the form of a Gaussian distribution as in Eq. (2) and accounts for crystalline defects associated with low growth temperatures (antisites, vacancies, interstitials). The only variable related to this profile which impacts the shape of the model is the standard deviation (σ_G_), which will be referred to as the exciton broadening to prevent confusion with σ_1_ or σ_2_ which refer to the localised state distributions. Equation (3) shows the overall model which calculates the PL intensity at a given energy.

3 $${I}_{eff}\left(E\right)={\int }_{{E}_{min}}^{{E}_{max}}{E}_{x}\left(E,{E}_{0},{\sigma }_{G}\right)\times {N}_{LDOS}\left(E,{E}_{m},{E}_{mob},\gamma ,{\sigma }_{1},{\sigma }_{2}\right) \bullet d{E}_{0}$$

Fitting to the experimental data was performed in MATLAB after normalising the spectra to a unity maximum. The fit quality was evaluated on an individual spectrum basis as a root mean square error (RMSE) between the model output and experimental data over an appropriate wavelength range, limited by either background noise or the intensity dropping to 1% of the peak value. The quality of the overall model derived for each device was then assessed as the mean value of the RMSE from all excitation powers, with only the E_loc_ filling parameter being allowed to change at the different laser powers. The lowest excitation power was not used in the RMSE calculations for any of the devices but will be presented alongside the modelled data using an appropriate E_loc_ value. Error bars for the model parameters were calculated by allowing the fitting program to vary all the parameters in parallel and setting the RMSE limit to 20% above that of the absolute best fit.

## Results and discussion

After initial results in which all of the parameters were allowed to vary it was noticed that σ_1_ and σ_2_ tended to have consistent values and that the fitting program would increase E_mob_ to unrealistic values above the GaAs band edge in order to produce marginal improvements to the fit quality. It was therefore deemed necessary to constrain these parameters in order to make any observed trends in the other variables more reliable.

**Figure 2:**
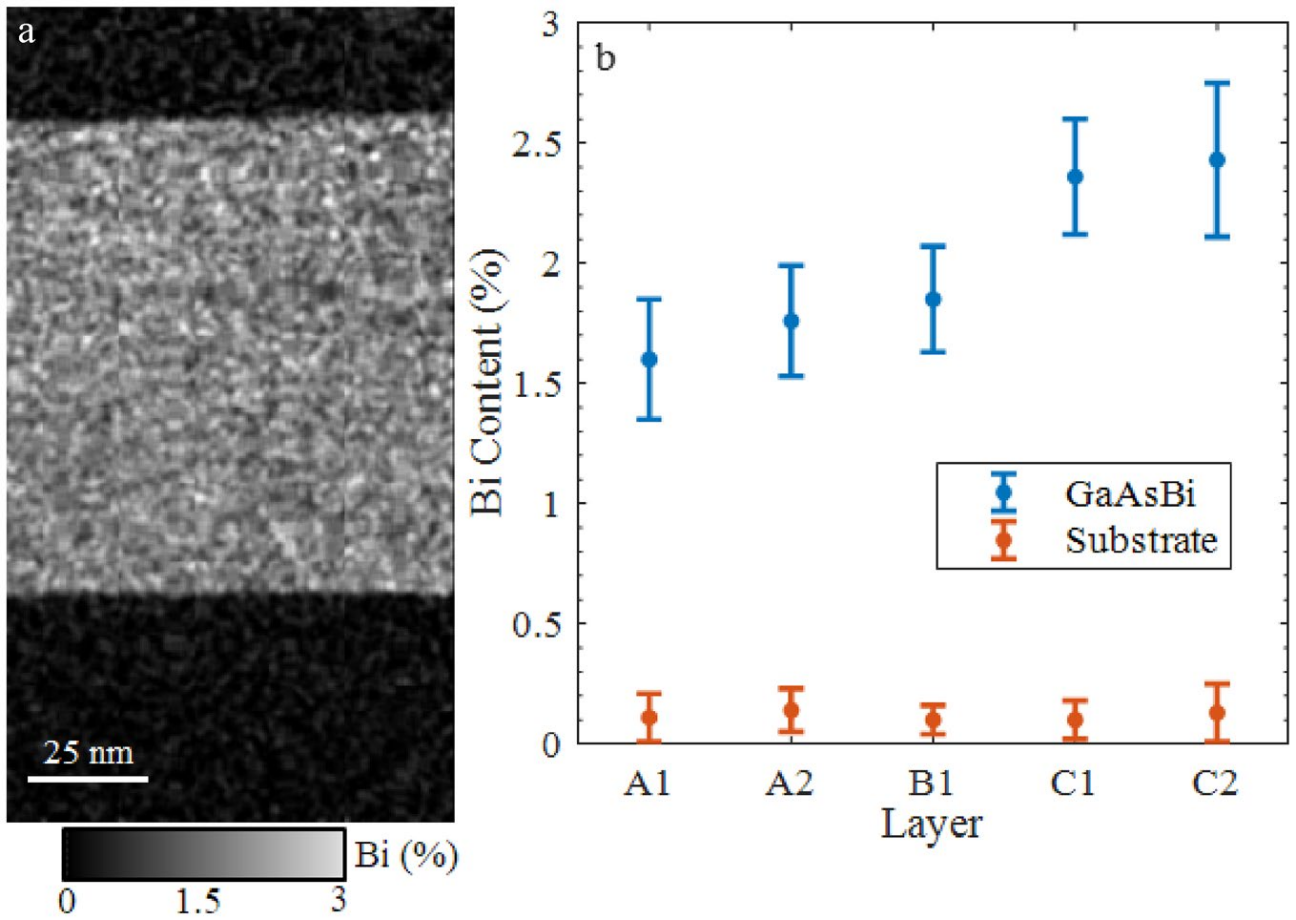
(**a**) EDX image of layer A1 and (**b**) Bi content in all control layers with standard deviation (error bars). Growth details for these layers are in the supplementary material.

The first parameter constrained was σ_1,_ which was fixed at the mean value of 27 meV. This is comparable to the standard deviation of the band gap in the GaAsBi control layers observed by STEM. Figure 2a shows an EDX Bi map for one of these control layers acquired at the [110] pole. In this map the darker contrast is associated with GaAs while the brighter area corresponds to the Bi layer. In the Bi layer, fluctuations in the Bi content can be clearly seen by the non-uniform brightness. These fluctuations of Bi observed by EDX have been analysed to give the average Bi contents and associated standard deviations (error bars) for all the control layers in Fig. 2b. As can be seen, the mean value in the substrate is close to zero with small fluctuations while in the layers it varies from 1.6 to 2.4. The standard deviation is 2 to 4 times higher in the layer than in the substrate, thus the variation in brightness can be ascribed to variation in composition to due fluctuations in Bi incorporation and not to measurement noise. Converting the Bi contents in Fig. 2b into energy^14^ produces deviation values ranging from 15.5 to 21 meV for the layers, which is within a factor of two of our estimation for σ_1_.

The mean value of σ_2_ was 96 meV which is close to the value of 100 meV found in^21^ where only a single Gaussian was used to fit the PL spectra. Also this is in broad agreement with the calculated energies to which the LDOS extends from other reports^15,16^.

**Figure 3:**
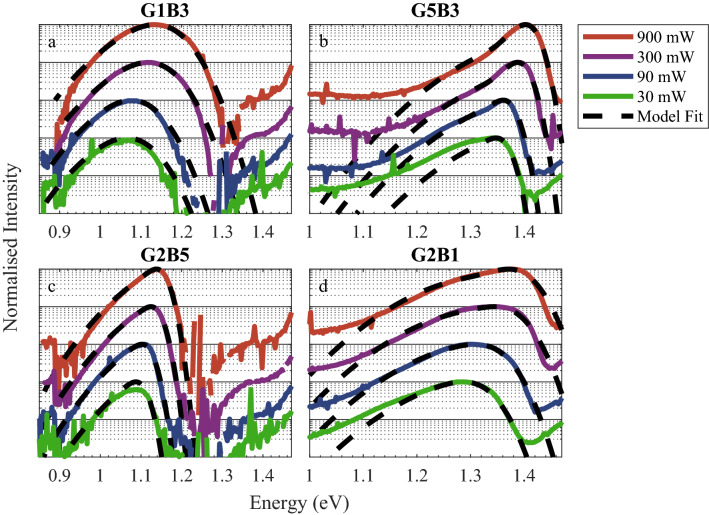
PL spectra at 30 K and model fits for devices grown at the limits of growth temperature and bismuth flux. (**a**) G1B3 grown at 355 °C and (**b**) G5B3 grown at 405 °C both under the same bismuth BEP of 1.06 × 10^–7^ mbar. (**c**) G2B5 grown under a bismuth BEP of 2.12 × 10^–7^ mbar and (**d**) G2B1 grown under a bismuth BEP of 0.5 × 10^–7^ mbar, both grown at 375 °C.

The final fits to the four devices which had the greatest difference in growth conditions are shown in Fig. 3 and the model parameters for all the devices are presented in Table 2. The devices grown at high temperature/BEP display a sharp drop-off in PL intensity at high energy whilst the low temperature/BEP devices are broader. Of all the devices studied, G1B3 exhibited the largest localisation energies, E_loc_, at all excitation powers, indicating very low state filling. This suggests a short non-radiative lifetime, as expected from the low growth temperature used for this device^29^. Due to this low level of carrier filling, the band edge state distribution, G_1_, contributed a negligible proportion of the filled states compared to G_2_ (equivalent to energies at and below 1.25 eV in Fig. 1) and is unobservable in any of the PL spectra for this device. As such the values of E_m_ and γ for this device are not credible and have been enclosed in parentheses is Table 2. It can be seen in Fig. 3b that there is a significant disparity between the modelled and raw data at 30 mW for sample G5B3. We believe this to be caused by two distinct bismuth compositions within the structure, as has been identified from XRD measurements for this device and G4B3 ^25^.

**Table 2:** Summary of best-fit model parameters for each device.

Layer	E mob (eV)	E m (eV)	E offset (eV)	σ_1_ (eV)	σ_2_ (eV)	σ_G_ (eV)	Gamma	RMSE
G1B3	1.276	(1.234)	(0.042)	0.027	0.096	0.046	(1)	0.0229
G2B3	1.271	1.242	0.029			0.033	0.17	0.0343
G3B3	1.319	1.353	− 0.034			0.02	0.43	0.0181
G4B3	1.361	1.401	− 0.040			0.018	0.4	0.0172
G5B3	1.419	1.456	− 0.037			0.015	0.26	0.0255
G2B1	1.422	1.351	0.071			0.031	0.18	0.0298
G2B2	1.33	1.303	0.027			0.035	0.28	0.0272
G2B4	1.242	1.274	− 0.032			0.022	0.37	0.0273
G2B5	1.172	1.25	− 0.078			0.017	0.7	0.0209

Localisation energies and the evaluation wavelength ranges can be found with the raw data in the supplementary material. The values for device G1B3 which are not considered accurate due to poor state filling are enclosed in parentheses.

**Figure 4:**
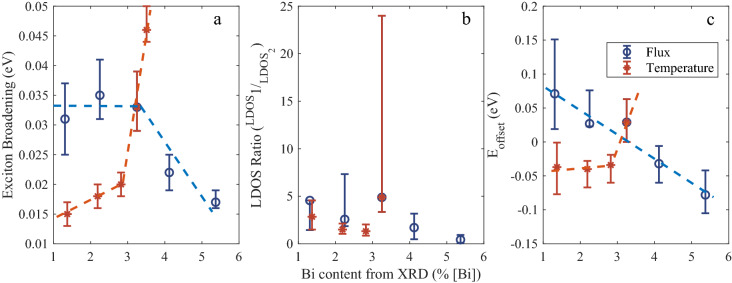
Trends in model parameters (**a**) σ_G_, (**b**) LDOS ratio and (**c**) E_offset_ versus Bi content from XRD modelling. Blue and orange dashed lines have been added to guide the eye.

Figure 4a–c show the trends of the exciton broadening, γ (represented as a ratio of the two distributions) and E_offset_ (taken as E_mob_ − E_m_) respectively as functions of Bi content. Device G1B3 has been omitted from Fig. 4b and c due to the uncertainty previously mentioned. The exciton broadening is seen to decrease rapidly with increasing growth temperature but also decreases with increasing bismuth BEP, indicating it is not solely dependent on the bismuth content of the layer. In Fig. 4a layers G5B3 and G4B3 are seen to have significantly lower exciton broadening values than G2B1 and G2B2 to fit their narrower PL spectra despite having similar Bi contents. This is in agreement with the findings in^26^ where an increase in growth temperature resulted in a reduction in FWHM for all layers. G1B3 displayed the largest exciton broadening of 46 meV which is believed to be caused by the low growth temperature. In Fig. 4b the LDOS ratio values all lie between 0.5 and 5 but show no clear trend although there appears to be a weak negative correlation with Bi content. E_offset_ in Fig. 4c broadly follows a similar trend to the exciton broadening except the three highest growth temperatures which have similar values.

**Figure 5:**
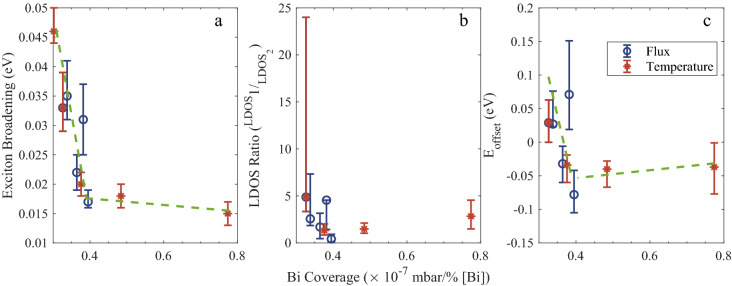
(**a**) σ_G_, (**b**) LDOS ratio and (**c**) E_offset_ plotted against Bi coverage calculated from bismuth content and BEP. Green dashed lines have been added to guide the eye.

In order to compare the temperature and BEP series in a more direct way a complimentary growth parameter was calculated for devices of both series. This was done by taking the Bi BEP supplied during growth and dividing it by the bismuth content extracted from XRD modelling. This value was referred to as the ‘Bi coverage’ as a low value would indicate efficient incorporation with little segregated Bi and a high value would indicate lower efficiency with more segregated Bi. Figure 5 shows the model parameters from Fig. 4 replotted against Bi coverage and it can be seen that both series follow remarkably similar trends where a higher ‘Bi coverage’ leads to reduced exciton broadening and narrower PL emission. It is worth noting that the layers in Fig. 5a with the lowest exciton broadening were also reported to have low saturation currents^25^. G5B3 and G4B3 had the lowest dark currents of all the devices and G2B5, despite containing over 5% Bi, had a dark current which appeared to only be increased due to the decrease in bandgap when compared to G5B3. This observation agrees with a report by Richards et al*.* which concludes that the Bi content does not impact the dark currents and that only the growth temperature and band gap affect this characteristic^29^. The overall findings of this work, however, indicate that surface Bi coverage does impact the material quality and may be essential in mitigating the deleterious effects of low temperature growth.

## Conclusions

In summary, a localised state filling model utilising two Gaussian distributions has been applied to approximate the LT PL from a series of GaAsBi devices grown using a variety of substrate temperatures and bismuth BEPs. It was found that good fits to the raw PL spectra could be achieved whilst constraining many of the parameters to physically justified values. Trends in the model variables indicate that both growth temperature and bismuth pressure impact the distribution of localised states through their combined influence on the bismuth surface coverage during growth. Comparison to the previously reported characteristics of these devices suggests that this model can be used to identify material which would display low dark currents and aid in reducing growth optimisation time.

## Supplementary Information

Supplementary Information 1.

Supplementary Information 2.

## References

[1] Sweeney, S. J., Batool, Z., Hild, K., Jin, S. R., & Hosea, T. J. C. The potential role of bismide alloys in future photonic devices. In 13th *International Conference on Transparent Optical Networks*, (IEEE) (2011).

[2] Marko IP, Sweeney SJ (2017). Progress toward III–V bismide alloys for near-and midinfrared laser diodes. IEEE J. Sel. Top. Quantum Electron..

[3] Thomas T (2015). Requirements for a GaAsBi 1 eV sub-cell in a GaAs-based multi-junction solar cell. Semicond. Sci. Technol..

[4] Richards, R. D., *et al*. GaAsBi: An alternative to InGaAs based multiple quantum well photovoltaics. In *43rd Photovoltaic Specialists Conference (PVSC)*, 1135–1137 (2016)

[5] Francoeur S (2003). Band gap of GaAs 1 − x Bi x, 0 < x < 3.6%. Appl. Phys. Lett..

[6] Hild K (2013). Auger recombination suppression and band alignment in GaAsBi/GaAs heterostructures. AIP Conf. Proc..

[7] Liu Y (2021). Valence band engineering of GaAsBi for low noise avalanche photodiodes. Nat. Commun..

[8] Young EC (2007). Bismuth incorporation in GaAs1–xBix grown by molecular beam epitaxy with in-situ light scattering. Phys. Status Solidi C.

[9] Tixier S (2003). Molecular beam epitaxy growth of GaAs 1–x Bi x. Appl. Phys. Lett..

[10] Feenstra RM, Woodall JM, Pettit GD (1993). Observation of bulk defects by scanning tunneling microscopy and spectroscopy: Arsenic antisite defects in GaAs. Phys. Rev. Lett..

[11] Krambrock K (1992). Arsenic antisite-related defects in low-temperature MBE grown GaAs. Semicond. Sci. Technol..

[12] Mohmad AR (2011). Photoluminescence investigation of high quality GaAs 1 – x Bi x on GaAs. Appl. Phys. Lett..

[13] Kudrawiec R (2009). Carrier localization in GaBiAs probed by photomodulated transmittance and photoluminescence. J. Appl. Phys..

[14] Mohmad AR (2014). Localization effects and band gap of GaAsBi alloys. Phys. Status Solidi B.

[15] Yoshimoto M (2013). Quantitative estimation of density of Bi-induced localized states in GaAs1 − xBix grown by molecular beam epitaxy. J. Cryst. Growth.

[16] Richards RD (2016). Telecommunication wavelength GaAsBi light emitting diodes. IET Optoelectron..

[17] Imhof S (2010). Clustering effects in Ga(AsBi). Appl. Phys. Lett..

[18] Valkovskii V, Jandieri K, Gebhard F, Baranovskii SD (2018). Rethinking the theoretical description of photoluminescence in compound semiconductors. J. Appl. Phys..

[19] Gogineni C (2013). Disorder and the Urbach edge in dilute bismide GaAsBi. Appl. Phys. Lett..

[20] Shakfa MK (2015). Thermal quenching of photoluminescence in Ga(AsBi). J. Appl. Phys..

[21] Wilson T (2018). Assessing the nature of the distribution of localised states in bulk GaAsBi. Sci. Rep..

[22] Yan B (2019). Bismuth-induced band-tail states in GaAsBi probed by photoluminescence. Appl. Phys. Lett..

[23] Riordan NA (2012). Temperature and pump power dependent photoluminescence characterization of MBE grown GaAsBi on GaAs. J. Mater. Sci. Mater. Electron..

[24] Bahrami-Yekta V, Tiedje T, Masnadi-Shirazi M (2015). MBE growth optimization for GaAs1 − xBix and dependence of photoluminescence on growth temperature. Semicond. Sci. Technol..

[25] Rockett TBO (2017). Influence of growth conditions on the structural and opto-electronic quality of GaAsBi. J. Cryst. Growth.

[26] Kakuyama K (2019). Impact of a small change in growth temperature on the tail states of GaAsBi. J. Appl. Phys..

[27] Richards RD (2014). Molecular beam epitaxy growth of GaAsBi using As2 and As4. J. Cryst. Growth.

[28] Mohmad, A. R., *et al.* Photoluminescence from localized states in GaAsBi epilayers. *2014 IEEE International Conference on Semiconductor Electronics (ICSE2014)*. IEEE (2014)

[29] Richards RD (2021). Temperature and band gap dependence of GaAsBi pin diode current–voltage behaviour. J. Phys. D.

