# Supplementary material for: Effect of MBE growth conditions on GaAsBi photoluminescence lineshape and localised state filling
Source: Sci Rep. 2022 Jan 17;12:797. doi: 10.1038/s41598-021-04477-0 (PMC8763887; doi:10.1038/s41598-021-04477-0)
Supplement: Supplementary file 2 — Supplementary Information 2. [file 41598_2021_4477_MOESM2_ESM.pdf]

Effect of MBE growth conditions on GaAsBi photoluminescence lineshape and localised state filling

N. J. Bailey[a], T. B. O. Rockett[a], S. Flores[b], D. F. Reyes[b], J. P. R. David[a], R. D. Richards[a*]

a) Department of Electronic and Electrical Engineering, University of Sheffield, United Kingdom
b) University Research Institute on Electron Microscopy & Materials, (IMEYMAT), Universidad de Cádiz, 11510 Puerto Real, Cádiz, Spain

CONTROL LAYER INFORMATION

The control layers studied by energy dispersive x-ray spectroscopy were grown in same Omicron MBE-STM reactor as the device layers studied in this work. The native oxide was desorbed at 610˚C for 30 min under an As_2 flux after which the substrate temperature was dropped to 570˚C for a 200 nm GaAs buffer using an As_2:Ga atomic flux ratio of 1.6. The substrate was then dropped to the GaAsBi growth temperature and the As species switched to As_4. 100nm of GaAsBi was then deposited under As_4:Ga atomic flux ratio of 2:1 alongside the Bi flux noted below. Finally a GaAs cap was deposited at 570˚C using the same As_2 flux.

Layer	GaAsBi growth temperature (˚C)	GaAsBi thickness (nm)	Bi flux ($\times 10^{-7}$ mbar)	Bi content (STEM) (%)
A1	375	100	0.705	1.6
A2	375	100	0.705	1.76
B1	360	100	0.545	1.85
C1	360	100	0.545	2.36
C2	360	100	0.545	2.43

Supplementary Table S1: Growth condition and measured Bi content of control layers.

ALL FITTING PARAMETERS

Table S2 below contains all the fitting parameters used for each layer including the localisation energies required to fit each excitation power (900 – 30 mW in order). Variables for G1B3 which are not considered reliable as noted in the manuscript are enclosed in parentheses.

Layer	Emob (eV)	Em (eV)	Sig 1 (eV)	Sig 2 (eV)	Ex (eV)	Gamma (0-1)	Eloc 1 (eV)	Eloc 2 (eV)	Eloc 3 (eV)	Eloc 4 (eV)
G1B3*	1.276	(1.234)			0.046	(1)	0.099	0.12	0.166	0.185
G1B3	1.277	(1.215)			0.047	(0.11)	0.093	0.114	0.16	0.179
G2B3*	1.271	1.242			0.033	0.17	0.018	0.029	0.06	0.088
G2B3	1.265	1.228			0.033	0.21	0.011	0.022	0.053	0.079
G3B3	1.319	1.353			0.02	0.43	0.001	0.018	0.039	0.061
G4B3	1.361	1.401			0.018	0.4	0	0.017	0.035	0.062
G5B3	1.419	1.456	0.027	0.096	0.015	0.26	0.001	0.018	0.044	0.056
G2B1	1.422	1.351			0.031	0.18	0.024	0.041	0.068	0.096
G2B2	1.330	1.303			0.035	0.28	0.011	0.025	0.055	0.077
G2B4*	1.242	1.274			0.022	0.37	0.021	0.033	0.06	0.078
G2B4	1.242	1.266			0.022	0.34	0.021	0.032	0.059	0.08
G2B5*	1.172	1.25			0.017	0.7	0.015	0.028	0.05	0.065
G2B5	1.161	1.219			0.019	0.52	0.004	0.018	0.039	0.056

*Supplementary Table S2: Complete set of model variables used to fit the GaAsBi device layers.*fits to data where substrate luminescence has been removed.*

<u>RAW AND FIT DATA FOR EACH LAYER</u>

The attached excel sheet (SM PL raw data and fits.xlsx) contains the raw and fit data for all the device layers at excitation powers of 30, 90, 300 and 900 mW at 33% duty cycle taken at 30 K. Emission from the GaAs substrate has been removed for some of the layers to allow proper scaling relative to the GaAsBi peak in the modelling program. The highlighted cells indicate the wavelength range which was used for modelling each excitation power for each layer (900 – 90 mW).
